# The Guanine Nucleotide Exchange Factor RIC8 Regulates Conidial Germination through Gα Proteins in *Neurospora crassa*

**DOI:** 10.1371/journal.pone.0048026

**Published:** 2012-10-31

**Authors:** Carla J. Eaton, Ilva E. Cabrera, Jacqueline A. Servin, Sara J. Wright, Murray P. Cox, Katherine A. Borkovich

**Affiliations:** 1 Department of Plant Pathology and Microbiology and Institute for Integrative Genome Biology, University of California Riverside, Riverside, California, United States of America; 2 Institute of Molecular BioSciences, Massey University, The Bio-Protection Research Centre and The Allan Wilson Centre for Molecular Ecology and Evolution, Palmerston North, New Zealand; Oregon State University, United States of America

## Abstract

Heterotrimeric G protein signaling is essential for normal hyphal growth in the filamentous fungus *Neurospora crassa*. We have previously demonstrated that the non-receptor guanine nucleotide exchange factor RIC8 acts upstream of the Gα proteins GNA-1 and GNA-3 to regulate hyphal extension. Here we demonstrate that regulation of hyphal extension results at least in part, from an important role in control of asexual spore (conidia) germination. Loss of GNA-3 leads to a drastic reduction in conidial germination, which is exacerbated in the absence of GNA-1. Mutation of RIC8 leads to a reduction in germination similar to that in the Δ*gna-1*, Δ*gna-3* double mutant, suggesting that RIC8 regulates conidial germination through both GNA-1 and GNA-3. Support for a more significant role for GNA-3 is indicated by the observation that expression of a GTPase-deficient, constitutively active *gna-3* allele in the Δ*ric8* mutant leads to a significant increase in conidial germination. Localization of the three Gα proteins during conidial germination was probed through analysis of cells expressing fluorescently tagged proteins. Functional TagRFP fusions of each of the three Gα subunits were constructed through insertion of TagRFP in a conserved loop region of the Gα subunits. The results demonstrated that GNA-1 localizes to the plasma membrane and vacuoles, and also to septa throughout conidial germination. GNA-2 and GNA-3 localize to both the plasma membrane and vacuoles during early germination, but are then found in intracellular vacuoles later during hyphal outgrowth.

## Introduction

Since the discovery of the first heterotrimeric G protein in filamentous fungi in the 1990's [1], G proteins have been found to play key roles in diverse fungal processes ranging from asexual and sexual development to pathogenicity of animal and phytopathogenic fungi (reviewed in Li et al, 2007). Most fungi possess three Gα subunits and a single Gβ and Gγ protein, therefore allowing for the assembly of three different heterotrimers. These three Gα subunits can act independently to regulate separate pathways, leading to differing phenotypes for single Gα mutants. For example, *Neurospora crassa* GNA-1 is required for normal vegetative growth, aerial hyphae formation and female fertility [2], whereas GNA-3 is required for normal production of asexual spores (conidia) and maturation of sexual spores (ascospores) [3]. In contrast, the *N. crassa* Δ*gna-2* mutant displays only a mild phenotype during growth on poor carbon sources [4]. However, loss of GNA-2 exacerbates phenotypes of the Δ*gna-1* and Δ*gna-3* mutants, indicating that GNA-2 shares overlapping functions with the other two Gα subunits [5], [6]. Indeed, all three G proteins are thought to act together to regulate certain processes, as mutants lacking GNA-1 and GNA-3 or all three Gα subunits are severely impaired in growth on solid medium, inappropriately conidiate in submerged liquid culture and do not produce female reproductive structures [6].

G protein coupled receptors (GPCRs), act as guanine nucleotide exchange factors (GEFs) for Gα subunits, facilitating exchange of GDP for GTP, thereby leading to activation and dissociation from the Gβγ dimer (reviewed in Li et al, 2007). However, recently a non-receptor GEF capable of activating Gα proteins, RIC8, has been identified in both animals and some fungi [7], [8], [9]. In *N. crassa*, loss of *ric8* leads to a severe growth impairment phenotype similar to that in mutants lacking both *gna-1* and *gna-3* or all three Gα subunit genes [9]. Expression of GTPase-deficient *gna-1* or *gna-3* alleles rescued many of the defects of the Δ*ric8* mutant during asexual growth on solid medium, and biochemical analyses showed that RIC8 can act as a GEF for both GNA-1 and GNA-3 *in vitro*, suggesting RIC8 acts upstream of both GNA-1 and GNA-3, particularly during regulation of asexual growth on solid medium [9]. Asexual hyphal growth is important for nutrient scavenging and for the organism to spread throughout the environment. In addition, it is important for encountering a mate of the opposite mating type, which allows the sexual cycle to proceed and produce the environmentally resistant sexual spores (ascospores).

Using a strain expressing a functional RIC8-GFP fusion, we have previously shown that RIC8 is a cytoplasmic protein [9]. Production of Gα-fluorescent protein fusions is problematic, as an N or C-terminal tag can interfere with normal functioning of the Gα protein. However, *Dictyostelium discoideum* Gα2 and mammalian Gα_o_ have been successfully tagged by insertion of GFP in a fold where it does not interfere with Gα_o_ function [10], [11].

In this study we further probe the role of RIC8, GNA-1 and GNA-3 in asexual hyphal growth and development. We analyze conidial morphology and determine conidial germination rates in *ric8* and G protein subunit mutants and in Δ*ric8* strains carrying GTPase-deficient alleles of *gna-1* or *gna-3*. We produce strains expressing GNA-1, GNA-2 and GNA-3 proteins as internal TagRFP fusions. We present here the first use of this internal tagging method for localization of Gα proteins in filamentous fungi. Using these strains, we determine the localization pattern of Gα proteins in conidia and during conidial germination and test for colocalization of RIC8 with GNA-1 and GNA-3.

## Materials and Methods

### Strains and growth conditions

*Neurospora* strains used in this study are listed in Table 1. For vegetative growth analysis, strains were grown on Vogel's minimal medium (VM; [12]). To induce the formation of female structures (protoperithecia) required for sexual crossing strains were grown on synthetic crossing medium (SCM; [13]). Cultures were inoculated with conidia and grown as described previously [4], [14].

**Table 1:** Strains used in this study.

Strain	Relevant genotype	Comments	Source
74-OR23-IVA	Wild type, *mat A*	FGSC 2489^a^	FGSC
r81-5a	Δ*ric8::hph^+^ mat a*	Δ*ric8* mutant	[9]
3B10	Δ*gna-1::hph^+^ mat a*	Δ*gna-1* mutant	[38]
FGSC 12378	Δ*gna-2::hph^+^ mat a*	Δ*gna-2* mutant	FGSC
31c2	Δ*gna-3::hph^+^ mat A*	Δ*gna-3* mutant	[3]
g1.3	Δ*gna-1::hph^+^* Δ*gna-3::hph^+^ mat a*	Δ*gna-1* Δ*gna-3* double mutant	[6]
noa	Δ*gna-1::hph^+^* Δ*gna-2::pyrG^+^*Δ*gna-3::hph^+^ mat A*	Δ*gna-1* Δ*gna-2* Δ*gna-3* triple mutant	[6]
R81*	Δ*ric8::hph^+^ gna-1* ^Q204L^ *::his-3^+^ mat A*	*gna-1* ^Q204L^ in Δ*ric8* background	[9]
R82*	Δ*ric8::hph^+^ gna-2* ^Q205L^ *::his-3^+^ mat A*	*gna-2* ^Q205L^ in Δ*ric8* background	[9]
R83*	Δ*ric8::hph^+^ gna-3* ^Q208L^ *::his-3^+^ mat A*	*gna-3* ^Q208L^ in Δ*ric8* background	[9]
42-8-3	Δ*gnb-1*, *mat A*	Δ*gnb-1* mutant	[43]
5-5-3	Δ*gng-1*, *mat A*	Δ*gng-1* mutant	[18]
Δ1his3	Δ*gna-1::hph^+^, his-3, mat A*		This Study
Δ2his3	Δ*gna-2::pyrG^+^, his-3, mat a*		This Study
31 h	Δ*gna-3::hph^+^, his-3, mat A*		This Study
2-1	Δ*gna-1::hph^+^, gna-1-TagRFP::his-3^+^ mat A*	Expresses GNA-1-TagRFP	This Study
5-1	Δ*gna-2::hph^+^, gna-2*-*TagRFP::his-3^+^ mat a*	Expresses GNA-2-TagRFP	This Study
12-1	Δ*gna-3::hph^+^, gna-3*-*TagRFP::his-3^+^ mat A*	Expresses GNA-3-TagRFP	This Study
R8GFP	Δ*ric8::hph^+^, ric8-GFP::his-3^+^, mat a*	Expresses RIC8-GFP	[9]

a FGSC, Fungal Genetics Stock Center, Kansas City, MO.

### Gα-TagRFP strain construction

**Figure 1:**
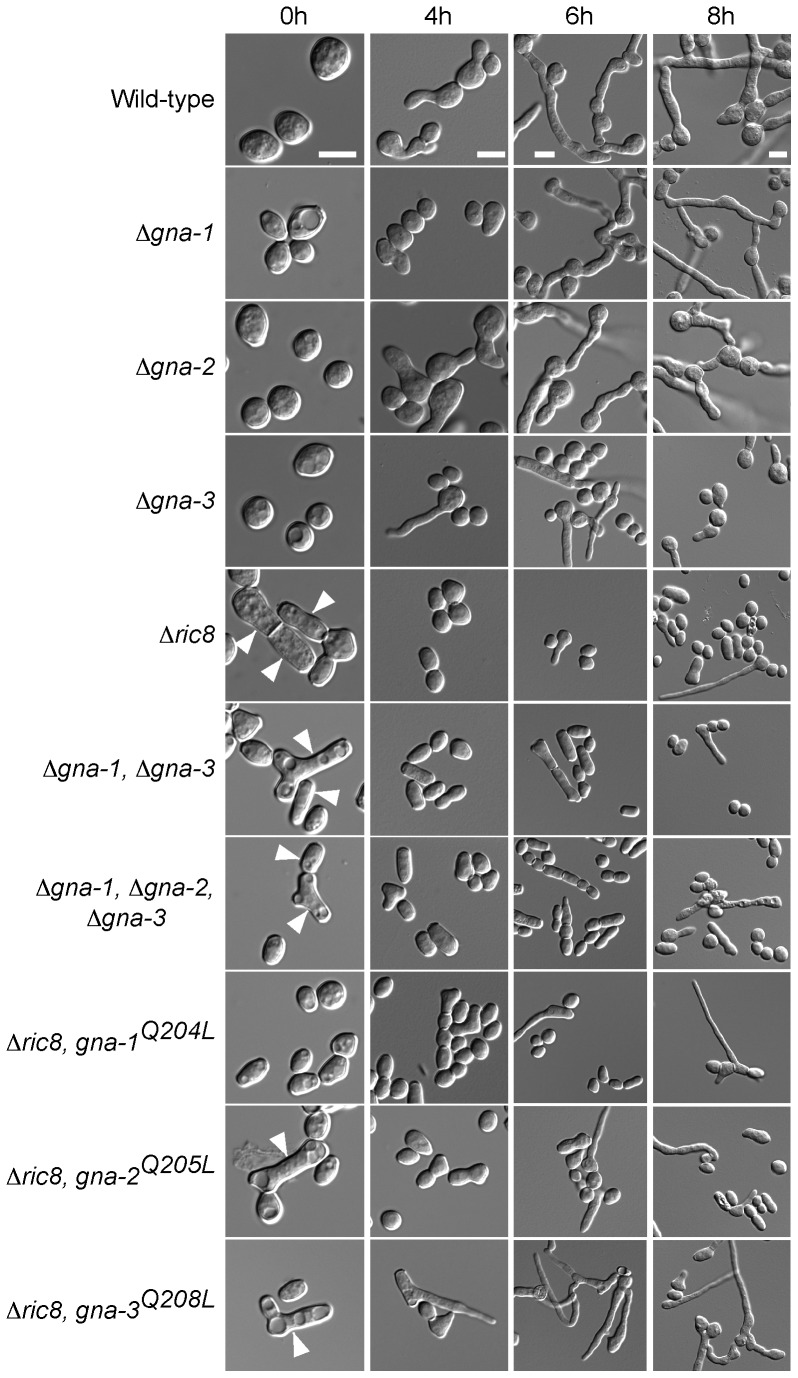
Alignment of Gα proteins. Amino acid alignment of *N. crassa* GNA-1, GNA-2, GNA-3 and chinese hamster GNAO1 (Genbank accession number ABA77543.1), showing position of the conserved loop into which TagRFP was inserted.

To observe the cellular localization of GNA-1, GNA-2 and GNA-3, TagRFP [15] was inserted into a conserved loop region within the Gα protein. This conserved loop region was found to be optimal for insertion of tags with conservation of Gα protein function in *Dictyostelium discoideum* Gα2 [11] and chinese hamster Gnao [10]; see Fig. 1). Primers were designed to prepare the Gα-TagRFP fusion constructs using yeast recombinational cloning, and are listed in Table 2. TagRFP was amplified from pAL3-Lifeact [15]; provided by Nick Read, University of Edinburgh) and the appropriate Gα N- and C-terminal fragments were amplified from cDNA clones. These fragments were inserted into pRS426 using yeast recombinational cloning [16]. The Gα-TagRFP fusion construct was then subcloned from pRS426 into pMF272 [17] as an *Eco*RI/*Xba*I fragment, resulting in replacement of *sgfp* from pMF272 with the Gα-TagRFP fusion and placing it under the control of the *ccg-1* promoter. The fusion constructs were then transformed into *his-3* Δ*gna-1*, Δ*gna-2* or Δ*gna-3* gene replacement mutants (See Table 1). Transformants were then screened by Southern blotting to ensure correct integration of the construct (data not shown).

**Table 2:** Primers used in this study.

Name	Anneals to	Sequence (5′–3′)
pRS426g1-F1	pRS426/N′ gna-1	ACGCCAGGGTTTTCCCAGTCACGACTCTAGAATGGGTTGCGGAATGAGTACAGAGGAG
g1TagRFP-R1	N′ gna-1/TagRFP	GCATGTTCTCCTTAATCAGCTCGCTCATCTCTAGGGACTCCATGGCCTCGAGAATG
g1TagRFP-F1	TagRFP/C′ gna-1	CGACCTCCCTAGCAAACTGGGGCACAAGTTGCCACTCGCCGATCAGCGCGTCGAG
pRS426g1-R1	C′ gna-1/pRS426	GCGGATAACAATTTCACACAGGAAACAGCGAATTCTCAAATCAAACCGCAGAGACGCAGG
TagRFP-F	TagRFP	ATGAGCGAGCTGATTAAGGAG
TagRFP-R	TagRFP	CTTGTGCCCCAGTTTGCTAGG
pRS426g3-F1	pRS426/N′ gna-3	GTAACGCCAGGGTTTTCCCAGTCACGACTCTAGAATGGGCGCATGCATGAGCAAGAACG
g3TagRFP-R1	N′ gna-3/TagRFP	GCATGTTCTCCTTAATCAGCTCGCTCATATCAAACTGGTGCATAGCATTCACAAC
g3TagRFP-F1	TagRFP/C′ gna-3	GCGACCTCCCTAGCAAACTGGGGCACAAGATCCAGCCAGCAGATCCGTCGCTACGG
pRS426g3-R1	C′ gna-3/pRS426	CGGATAACAATTTCACACAGGAAACAGCGAATTCTCATAGAATACCGGAGTCTTTAAGGG
pRS426g2-F1	pRS426/N′ gna-2	GTAACGCCAGGGTTTTCCCAGTCACGACTCTAGAATGTGTTTCGGGGGTCGTGGAAAGG
g2TagRFP-R1	N′ gna-2/TagRFP	GCATGTTCTCCTTAATCAGCTCGCTCATGTTGAACTCATTCATCGCATCAAAGATC
g2TagRFP-F1	TagRFP/C′ gna-2	GCGACCTCCCTAGCAAACTGGGGCACAAGATCAAGCTGGAGGATGAAGATAATGAG
pRS426g2-R1	C′ gna-2/pRS426	GGATAACAATTTCACACAGGAAACAGCGAATTCCTACAGGATAAGTTGTTTCAGGTTTCG
TagRFP-F2	TagRFP	TCTAGAATGAGCGAGCTGATTAAGGAG
TagRFP-R2	TagRFP	GAATTCTCACTTGTGCCCCAGTTTGC

### Western blot analysis

Western blotting was used to detect Gα-TagRFP fusion proteins in whole-cell extracts prepared from macroconidia. Conidia from 6–7 day flask cultures were collected using sterile water, pelleted and stored at −80°C. After thawing on ice, the conidia were resuspended using 1 ml of extraction buffer (10 mM HEPES, pH 7.5, 0.5 mM EDTA, 0.5 mM PMSF, 1 mM DTT and 0.1% v/v of fungal protease inhibitor (Sigma-Aldrich, St. Louis, MO; Product #T8215) and then transferred to a mortar. The conidia were vigorously crushed using a mortar and pestle under liquid nitrogen. Roughly equal amounts of ground tissue were then transferred to 2 ml screw cap tubes, and topped off with additional extraction buffer if necessary. Samples were spun at 3,300 RPM (1,000×g) for 10 minutes at 4°C in a microcentrifuge and the supernatants (whole cell extracts) retained.

Protein concentration was determined using the Bradford Protein Assay (Bio-Rad, Hercules, CA). Samples containing 50 µg of whole-cell protein were subjected to SDS-PAGE using 10% gels and gels transferred to nitrocellulose membranes as described previously [18]. Western analysis was performed using a polyclonal RFP antibody (#R10367, Invitrogen, Carlsbad, CA) as the primary antibody at a dilution of 1∶2000. A horseradish peroxidase conjugate antibody (Bio-Rad) was used as the secondary antibody at a dilution of 1∶5000 and chemiluminescent detection was performed as described previously [18].

### Microscopy

For analysis of conidial germination, 8×10^6^ conidia were spread on 100 mm 10 ml VM agarose plates and incubated at 30°C for 0, 4, 6 or 8 h. Cells were then visualized using differential interference contrast (DIC) microscopy using an Olympus IX71 inverted microscope (Olympus America, Center Valley, PA) with a 60× oil immersion objective (NA = 1.42). Images were captured using a QIClickTM digital CCD camera (QImaging, Surrey, British Columbia, Canada) and analyzed using Metamorph software (Molecular Devices Corporation, Sunnyvale, CA). For analysis of conidial anastomosis tubes, conidia were spread on VM agarose, and imaged after 5–16 h at 30°C as detailed above.

For observation of Gα localization, VM agarose plate cultures were prepared as described above. Germinating conidia were analyzed using a Leica TCS SP5 II confocal microscope with a 63× oil objective (NA =  1.40; Leica Microsystems Inc., Buffalo Grove, IL). The Gα-RFP strains were visualized with the Hybrid Detection system (HyD) laser at an excitation of 543 nm, and emission of 565–665 nm.

To confirm vacuolar localization, conidia from the Gα-TagRFP strains were inoculated onto VM agarose plates and incubated as described above. An aliquot containing 30 µl of a 20 µg/ml solution of Oregon Green 488 carboxylic acid diacetate (Carboxy-DFFDA; catalog number O6151; Molecular Probes) was applied to a coverslip. An agarose block containing germinating conidia was inverted onto the coverslip and incubated for 5 minutes at room temperature in the dark. Images were obtained using the Leica TCS SP5 II confocal microscope described above. GFP images were obtained by excitation at 488 nm, with emission collected from 500–535 nm. RFP images were obtained with excitation at 543 nm and emission from 555–700 nm. Images were captured sequentially in order to prevent crosstalk among samples.

The vacuolar and plasma membrane localization of GNA-1-TagRFP was further explored by imaging a heterokaryon that expresses GNA-1-TagRFP and a GFP fusion of the Ca^2+^ ATPase, NCA-3 [19]. NCA-3-GFP is known to localize to vacuoles and the plasma membrane [19]. The two strains were co-inoculated onto a VM slant in order to produce a heterokaryon with conidia expressing both fluorescent proteins. Conidia were inoculated onto VM agarose plates as described above and imaged 6 h later using a 543 nm HyD laser and 488 nm laser on the Leica TCS SP5 II confocal microscope. Images were captured sequentially.

Possible co-localization of RIC8 and GNA-1 or GNA-3 was investigated through co-culturing of the RIC8-GFP strain with the GNA-1-TagRFP or GNA-3-TagRFP strains on a VM slant to produce a heterokaryon expressing two fluorescent proteins. Conidia from the heterokaryons were inoculated onto VM agarose plates as described above, followed by incubation at 30°C for 0 or 6 h for GNA-1-TagRFP/RIC8-GFP and 0 or 4 h for the GNA-3-TagRFP/RIC8-GFP fusion strain. Images were obtained by confocal microscopy, as described above.

### Statistical analysis

Germination rates of strains relative to wild type were determined using Student's two-sided t test, with values paired by day of analysis [20]. Multiple comparisons within time points were corrected using the false discovery rate (FDR) approach of [21]. Differences in arthroconidiation and germination rates between strains were determined using Student's unpaired two-sided t test. Germination rates were first normalized for daily differences in wild type germination rates. All statistics were performed in R [22]; code is available on request. Details of the statistical analyses are presented in Tables 3, 4, 5, 6.

**Table 3:** Statistical analysis of arthroconidiation.

Strain 1	Strain 2	*t* a	*df* b	*p* c	sig^d^
*Δric8*	Wild type	**−8.02**	**2.18**	**0.024**	*
*Δgna-1Δgna-3*	”	**−11.02**	**2.18**	**0.018**	*
*Δgna-1Δgna-2Δgna-3*	”	**−20.41**	**2.81**	**0.002**	**
*Δric8gna1* *	*Δric8*	−0.19	3.51	0.991	ns
*Δric8gna2* *	”	−0.28	3.92	0.991	ns
*Δric8gna3* *	”	0.01	3.90	0.991	ns

a Student's t statistic,

b degrees of freedom,

c probability value, and ^d^ significance range, where ns is not significant;

* , 0.05≥*p*>0.01;

** , 0.01≥*p*>0.001. Statistically significant results are bolded.

**Table 4:** Statistical analysis of germination rates relative to wild type.

Strain 1	Strain 2	0 h				4 h				6 h				8 h			
		*t* a	*df* b	*p* c	sig^d^	*t*	*df*	*p*	sig	*t*	*df*	*p*	sig	*t*	*df*	*p*	sig
*Δgna-1*	Wild type	1.00	4	0.772	ns	1.94	4	0.140	ns	**3.77**	**4**	**0.027**	*	2.41	4	0.090	ns
*Δgna-2*	”	0.02	4	0.982	ns	1.92	4	0.140	ns	−0.52	4	0.627	ns	−1.41	4	0.254	ns
*Δgna-3*	”	0.15	4	0.982	ns	**5.59**	**4**	**0.008**	**	**7.91**	**4**	**0.003**	**	**5.63**	**4**	**0.009**	**
*Δric8*	”	−1.10	5	0.772	ns	**7.63**	**5**	**0.002**	**	**21.86**	**5**	**0.000**	***	**26.88**	**5**	**0.000**	***
*Δgna-1Δgna-3*	”	1.73	3	0.772	ns	**19.80**	**3**	**0.001**	***	**49.08**	**3**	**0.000**	***	**23.66**	**3**	**0.001**	***
*Δgna-1Δgna-2Δgna-3*	”	−0.06	3	0.982	ns	**22.73**	**3**	**0.001**	***	**21.47**	**3**	**0.001**	***	**36.33**	**3**	**0.000**	***
*Δric8gna1* *	”	1.73	3	0.772	ns	**21.36**	**3**	**0.001**	***	**24.97**	**3**	**0.001**	***	**9.71**	**3**	**0.005**	**
*Δric8gna2* *	”	1.00	2	0.772	ns	**16.80**	**2**	**0.006**	**	**14.89**	**2**	**0.007**	**	**13.34**	**2**	**0.009**	**
*Δric8gna3* *	”	−0.47	3	0.922	ns	**10.23**	**3**	**0.004**	**	**7.90**	**3**	**0.007**	**	**12.48**	**3**	**0.003**	**
*Δgnb1*	”	−0.84	2	0.772	ns	1.65	2	0.240	ns	1.32	2	0.349	ns	0.51	2	0.660	ns
*Δgng1*	”	−1.40	2	0.772	ns	**5.08**	**2**	**0.050**	*	1.95	2	0.233	ns	3.90	2	0.083	ns

a Student's t statistic,

b degrees of freedom,

c probability value, and ^d^ significance range, where ns is not significant;

* , 0.05≥*p*>0.01;

** , 0.01≥*p*>0.001;

*** , *p*≤0.001. Statistically significant results are bolded.

**Table 5:** Statistical analysis of germination rates between strains other than wild type.

Strain 1	Strain 2	0 h				4 h				6 h				8 h			
		*t* a	*df* b	*p* c	sig^d^	*t*	*df*	*p*	sig	*t*	*df*	*p*	sig	*t*	*df*	*p*	sig
*Δric8*	*Δric8gna1* *	−2.05	4.9	0.097	ns	−1.47	6.7	0.188	ns	0.39	7.9	0.710	ns	2.30	4.3	0.078	ns
*Δric8*	*Δric8gna2* *	−1.38	3.0	0.260	ns	−1.42	6.9	0.198	ns	−0.41	3.8	0.705	ns	−0.07	3.0	0.946	ns
*Δric8*	*Δric8gna3* *	0.21	3.3	0.847	ns	**3.60**	**6.3**	**0.011**	*	**13.06**	**8.0**	**0.000**	***	**14.34**	**8.0**	**0.000**	***
*Δric8*	*Δgna-3*	−0.67	6.4	0.527	ns	0.86	8.5	0.411	ns	1.79	5.6	0.128	ns	**3.18**	**4.9**	**0.025**	*
*Δric8*	*Δgna-1Δgna-3*	−2.05	4.9	0.097	ns	−1.77	7.0	0.119	ns	−1.81	7.0	0.114	ns	−1.22	6.6	0.263	ns
*Δgna-3*	*Δgna-1Δgna-3*	−1.12	6.9	0.301	ns	**−2.66**	**5.4**	**0.042**	*	**−2.80**	**4.4**	**0.044**	*	**−3.70**	**5.2**	**0.013**	*
*Δgna-1Δgna-3*	*Δgna-1Δgna-2Δgna-3*	1.04	5.4	0.343	ns	0.07	5.9	0.946	ns	0.17	4.1	0.877	ns	−0.64	5.3	0.551	ns

a Student's t statistic,

b degrees of freedom,

c probability value, and ^d^ significance range, where ns is not significant;

* , 0.05≥*p*>0.01;

** , 0.01≥*p*>0.001;

*** , *p*≤0.001. Statistically significant results are bolded.

**Table 6:** Statistical analysis of germination rates between wild type and Gα-TagRFP expressing strains.

Strain 1	Strain 2	0 h				4 h				6 h				8 h			
		*t* a	*df* b	*p* c	sig^d^	*t*	*df*	*p*	sig	*t*	*df*	*p*	sig	*t*	*df*	*p*	sig
*Δgna-1*/GNA-1-TagRFP	Wild type	−0.95	2	0.515	ns	−1.48	2	0.651	ns	−0.24	2	0.833	ns	−3.60	2	0.158	ns
*Δgna-2*/GNA-2-TagRFP	Wild type	0.82	2	0.515	ns	0.93	2	0.651	ns	3.29	2	0.122	ns	0.46	2	0.692	ns
*Δgna-3*/GNA-3-TagRFP	Wild type	0.78	2	0.515	ns	−0.53	2	0.651	ns	3.38	2	0.122	ns	2.83	2	0.158	ns

a Student's t statistic,

b degrees of freedom,

c probability value, and ^d^ significance range, where ns is not significant.

## Results

### Loss of *ric8* or both *gna-1* and *gna-3* leads to overproduction of arthroconidia

We have previously demonstrated that Δ*gna-1* and Δ*ric8* single mutants, Δ*gna-1* Δ*gna-3* double mutants, and the triple Gα mutant have smaller colony sizes than wild type [2], [6], [9]. The defects of the latter three mutants are similar and much more severe than those of the Δ*gna-1* strain [6], [9]. Smaller colony size could result from slow germination, reduced hyphal extension or both phenomena. In order to determine whether germination defects contribute to the overall reduction in colony size, we investigated early events during germination of conidia in Δ*ric8* mutants and in strains lacking G protein subunit genes.

**Figure 2:**
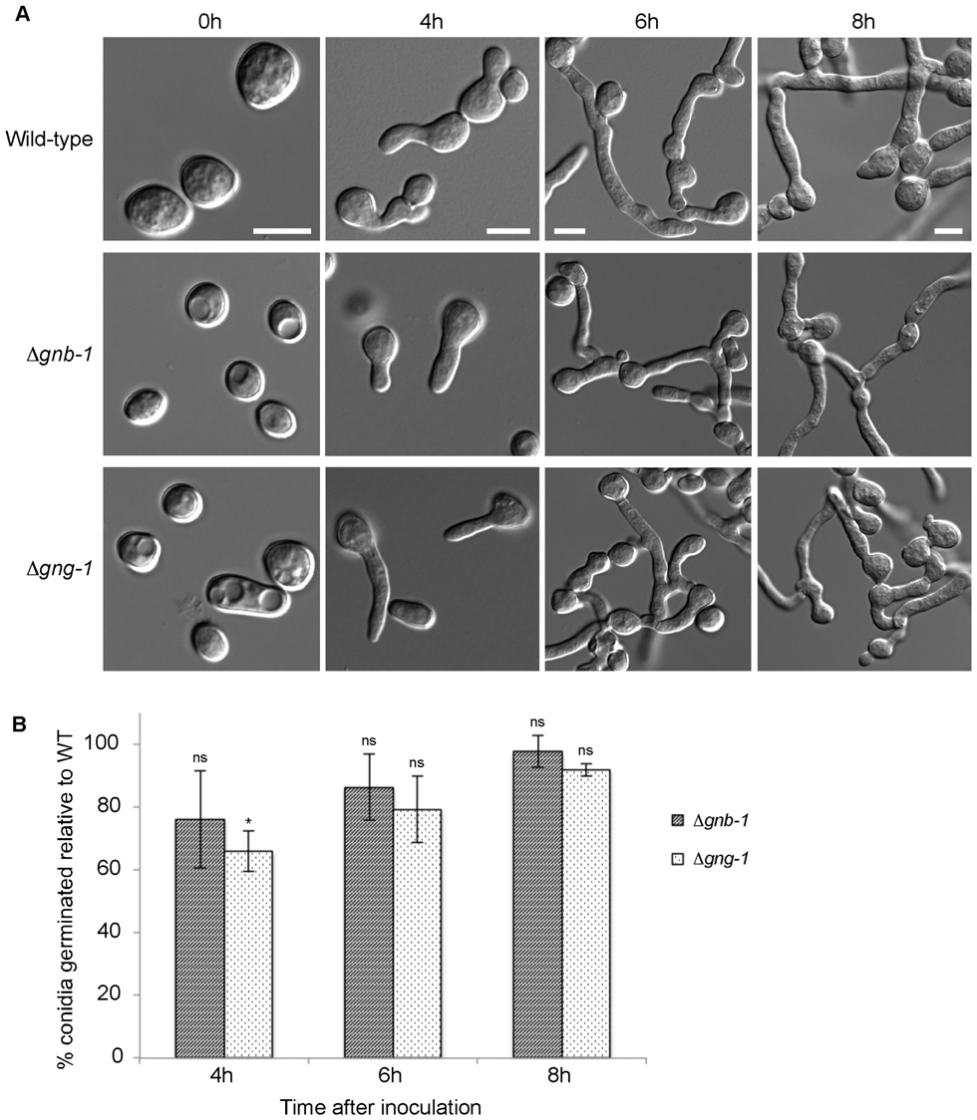
G proteins and the RIC8 GEF are required for normal conidiation and conidial germination. Conidia were harvested as described in the Materials and Methods. An aliquot containing 8×10^6^ conidia was spread on a 100 mm 10 ml VM solid medium plate and spore germination monitored at 30°C over time. DIC (differential interference contrast) micrograph images were obtained using an Olympus IX71 microscope with a QIClick digital CCD camera and analyzed using Metamorph software. Scale bar = 5 µm. Strains are wild type (74-OR23-IVA), Δ*gna-1* (3B10), Δ*gna-2* (FGSC 12378), Δ*gna-3* (31c2), Δ*ric8* (r81-5a), Δ*gna-1*, Δ*gna-3* (g1.3), Δ*gna-1*, Δ*gna-2*, Δ*gna-3* (noa), Δ*ric8, gna-1* ^Q204L^ (R81*), Δ*ric8, gna-2* ^Q205L^ (R82*), Δ*ric8, gna-3* ^Q208L^ (R83*). Arrowheads indicate arthroconidia.

**Figure 3:**
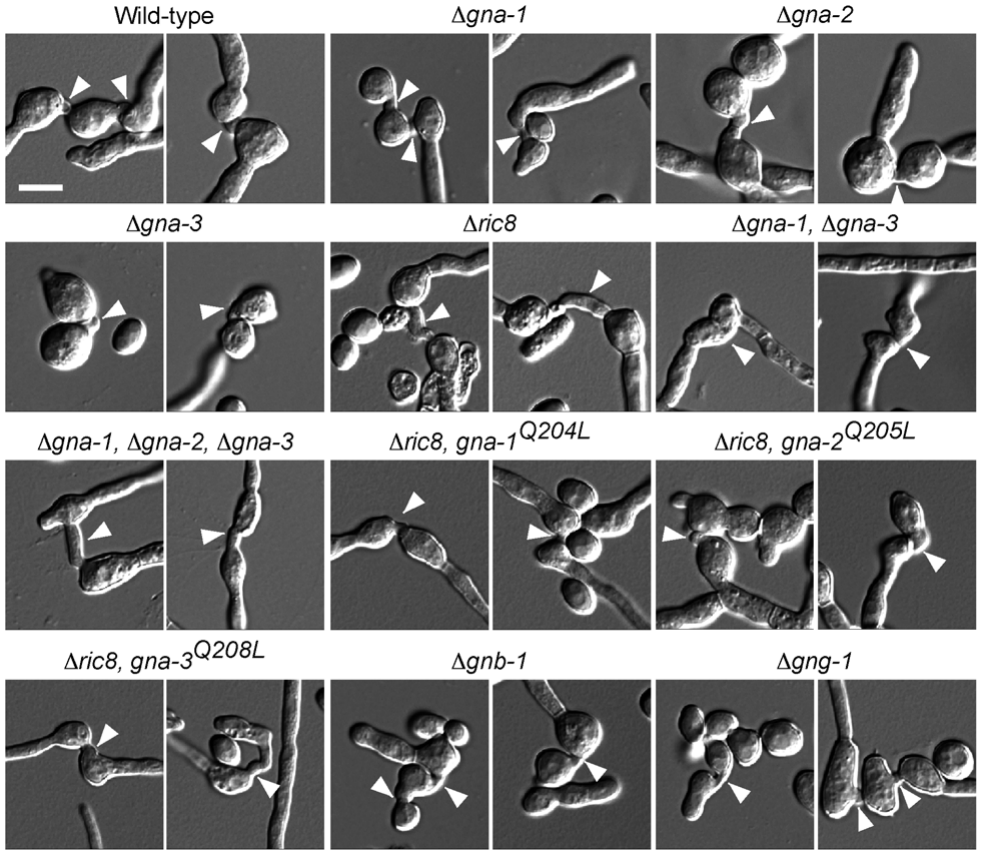
Germination of Δ*gnb-1* and Δ*gng-1* mutants. **A.** Conidia of wild-type (74-OR23-IVA), Δ*gnb-1* (42-8-3) and Δ*gng-1* (5-5-3) strains were harvested as described in the Materials and Methods. An aliquot containing 8×10^6^ conidia was spread on VM solid medium and germination monitored at 30°C over time. DIC images were obtained using an Olympus IX71 microscope with a QIClick™ digital CCD camera and analyzed using Metamorph software. Bar = 5 µm. **B**. Proportion of germinated conidia of *N. crassa* wild-type, Δ*gnb-1* and Δ*gng-1* strains at various times after inoculation onto VM medium. Error bars are ±SE for a minimum of three independent experiments (n = minimum of 100 cells for all strains). Strains are the same as in A.

We began with an analysis of conidial morphology. All five *N. crassa* G protein subunit mutants (Δ*gna-1*, Δ*gna-2*, Δ*gna-3*, Δ*gnb-1* and Δ*gng-1*) produce normal-looking macroconidia (Fig. 2; Fig. 3). However, Δ*ric8*, the Δ*gna-1* Δ*gna-3* double mutant and the triple Gα mutant all display an increased proportion of arthroconidia (wild type 6.2%±0.8%; Δ*ric8* 36.0%±3.6%; Δ*gna-1* Δ*gna-3* 47.1%±3.6%; and Δ*gna-1* Δ*gna-2* Δ*gna-3* 43.8%±1.7%) (Fig. 2, Table 3). Arthroconidia are formed by fragmentation of hyphae [23]. Introduction of GTPase-deficient alleles for any of the three Gα genes did not lead to a significant reduction in the proportion of arthroconidia in the Δ*ric8* background (Δ*ric8, gna-1* ^Q204L^ 36.8%±2.5%; Δ*ric8, gna-2* ^Q205L^ 37.6%±4.2%; Δ*ric8, gna-3* ^Q208L^ 36.0%±4.3%) (Fig. 2, Table 3). Therefore, inhibition of arthroconidiation depends on RIC8, GNA-1 and GNA-3, and constitutive activation of a Gα subunit is not sufficient to override the phenotype in Δ*ric8* mutants.

In *N. crassa*, arthroconidiation has been proposed to be the default conidiation pathway in mutants with defects in macroconidiation [24]. Arthroconidia are the major cell type used to disseminate many animal fungal pathogens, such as the valley fever fungus *Coccidioides immitis* and dermatophytes in the genera Mycosporum, Trichophyton and Epidermophyton [25], [26], [27]. To date, little is known about the regulation of arthroconidiation in these fungi, but it seems clear that RIC8, GNA-1 and GNA-3 impact this process in *N. crassa*.

### G protein signaling is required for conidial germination, but is not essential for conidial anastomosis tube formation

**Figure 4:**
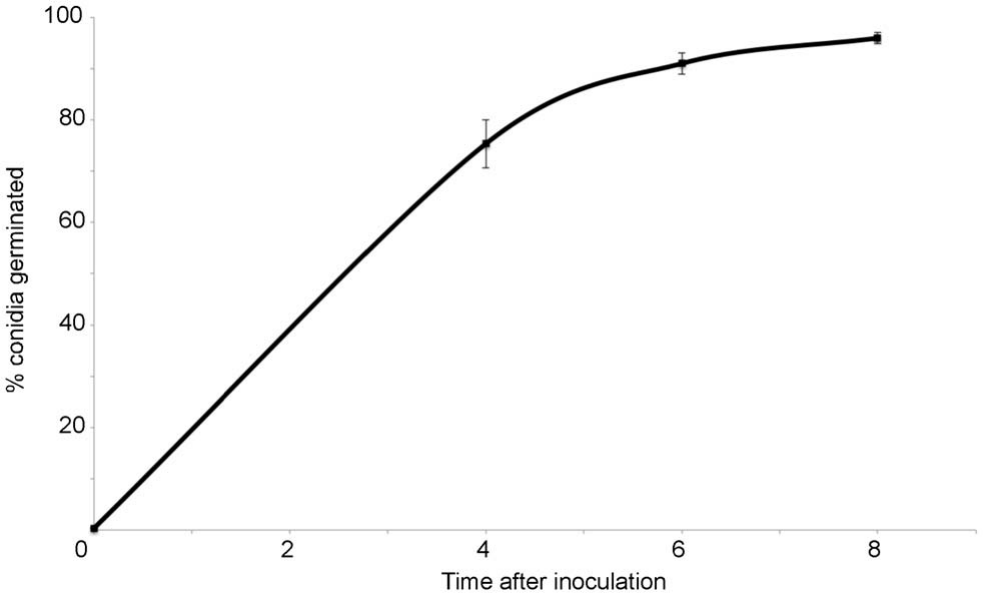
G protein mutants produce conidial anastomosis tubes (CATs). Conidia were used to inoculate 100 mm 10 ml solid VM agarose plates as described in Fig. 2. Plates were incubated at 30°C for 5–16 h depending on the germination rate of the mutant strain. Images were captured and analyzed and strains are the same as in Fig. 2. Arrowheads indicate positions of CATs. Bar = 5 µm.

We next investigated the ability of macroconidia from the various strains to form conidial anastomosis tubes (CATs) and to germinate on solid medium. CATs are small tube-like structures produced by conidia early during colony initiation (rev. in [28]). The results demonstrate that Δ*ric8* and all G protein subunit single and double mutants produce CATs from macroconidia (Fig. 4).

**Figure 5:**
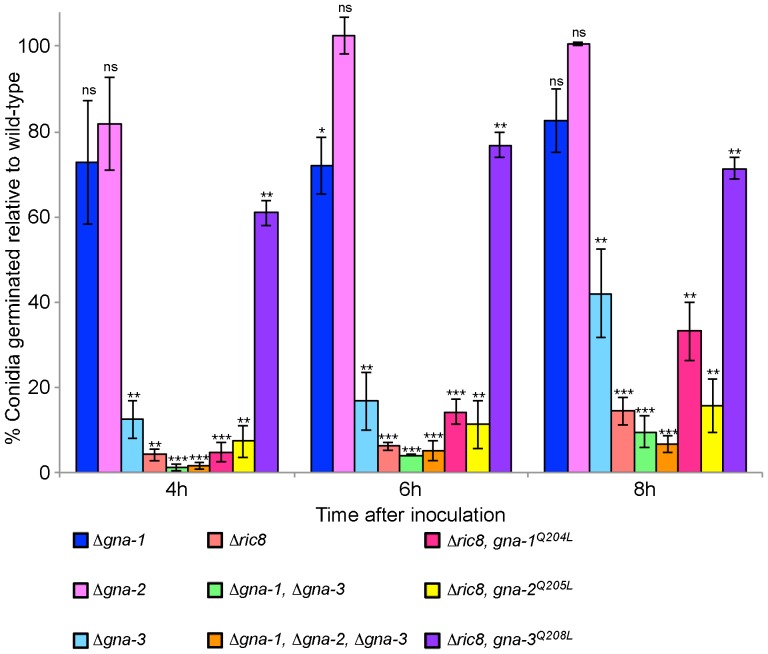
Germination rate of wild-type *N. crassa*. Values are taken from Table 7. Error bars are ±SE for twelve independent experiments (n = minimum of 60 cells).

**Figure 6:**
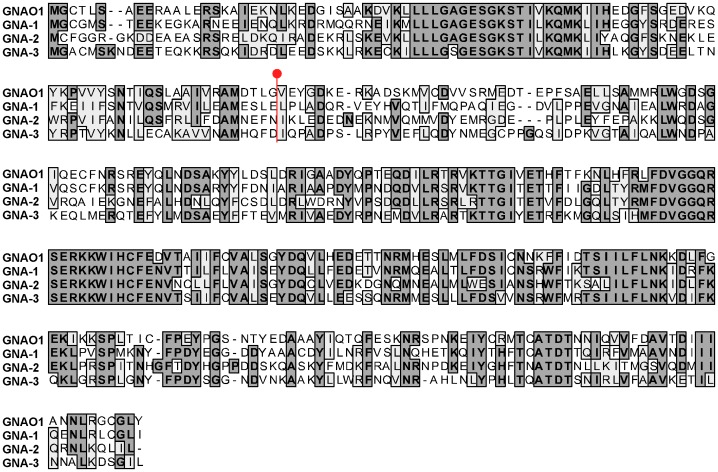
Quantitation of conidial germination rates in G protein and *ric8* mutants. The proportion of germinated conidia of wild-type, Gα mutant and Δ*ric8* strains was determined at various times after inoculation onto solid medium. Error bars are ±SE for a minimum of three independent experiments (n = minimum of 60 cells for all strains). Strains and conditions are the same as in Fig. 2.

We next analyzed conidial germination (both macroconidia and arthroconidia) in the various strains at 4, 6 and 8 h after plating on solid medium. In wild-type, greater than 70% of the conidia have germinated by 4 hr after inoculation, with nearly 100% germination after 8 h (Fig. 5; Table 7). In contrast, germination is significantly reduced in Δ*ric8*, Δ*gna-3*, Δ*gna-1* Δ*gna-3* and the triple Gα mutants relative to wild type at all time points (Fig. 2, 6). Germination of the Δ*gna-1* mutant also appears to be slower than wild-type, but this is only statistically significant at 6 h. However, the observation that germination of the Δ*gna-1* Δ*gna-3* mutant is significantly lower than the Δ*gna-3* mutant supports a compensatory role for GNA-1 in conidial germination (Fig. 2, 6). Loss of *gna-2* had no effect on conidial germination, and did not exacerbate the germination defect of the Δ*gna-1* Δ*gna-3* mutant (Fig. 2). Taken together, the results indicate that RIC8, GNA-3, and to a lesser extent GNA-1, play important roles during conidial germination.

**Table 7:** Germination rate of wild-type *N. crassa*.

	0 h	4 h	6 h	8 h
Average percentage of conidia germinated	0.4	75.3	91.0	96.0
Standard error	0.1	4.7	2.1	1.1

Subsequently, we tested whether constitutive activation of any of the three Gα subunits could rescue the conidial germination defect of Δ*ric8* strains. Introduction of the GTPase-deficient *gna-1* or *gna-2* alleles did not significantly improve germination, (Fig. 2, 6; Table 4, 5). Strikingly, constitutive activation of *gna-3* in the Δ*ric8* background resulted in a significant increase in conidial germination, approaching that of the wild-type strain (Fig. 6; Table 4, 5). The ability of the GTPase-deficient *gna-3* allele to bypass the Δ*ric8* germination defect supports RIC8 acting through GNA-3 to regulate conidial germination in *N. crassa*. We also probed a possible role for the Gβ and Gγ subunits during conidial germination (Fig. 3, Table 4). Similar to the results exploring conidial morphology, germination is largely unaffected in Δ*gnb-1* and Δ*gng-1* mutants, aside from a slight delay in Δ*gng-1* 4 h after inoculation.

### The three Gα proteins localize to the plasma membrane and vacuoles during conidial germination

Having demonstrated that Gα proteins are important for conidial germination, we next investigated the subcellular localization of these proteins during this process. For these experiments, we took advantage of previous studies in *Dictyostelium* and mammalian cells demonstrating that insertion of a fluorescent tag in a loop region of the Gα subunit does not disrupt the interaction with the Gβγ dimer or interfere with Gα function (Fig. 1; [10], [11].

We expressed all three Gα-TagRFP fusions under control of the highly expressed *ccg-1* promoter from the *his-3* locus in the corresponding Gα mutant strain (see Materials and Methods). Expression of GNA-1-TagRFP complemented the conidial germination (Table 6) and female fertility defects and partially complemented the growth rate phenotype of the Δ*gna-1* mutant (data not shown). Expression of GNA-3-TagRFP in the Δ*gna-3* mutant restored conidial germination back to wild-type levels at all time points investigated (Table 6).

**Figure 7:**
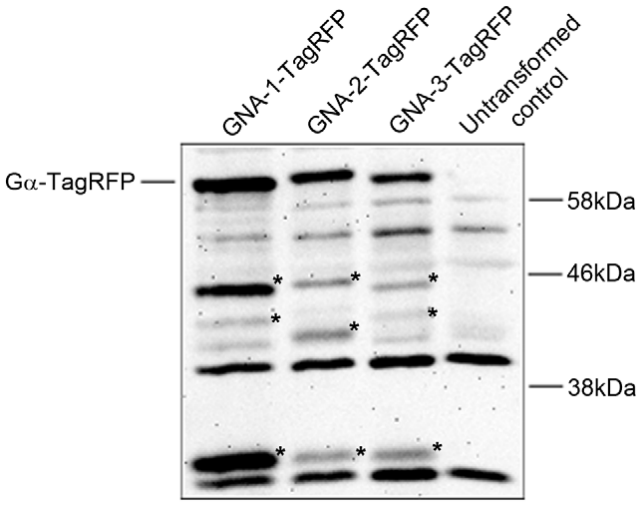
Western blot detection of Gα-TagRFP fusion proteins. Samples containing 50 µg of protein from conidial extracts were subjected to western blot analysis with a RFP primary antiserum as described in the Materials and Methods. Strains are Δ*gna-1*, *gna-1-TagRFP* (2.1), Δ*gna-2*, *gna-2-TagRFP* (5.1), Δ*gna-3*, *gna-3-TagRFP* (12.1) and wild type (untransformed control; 74-OR23-IVA). TagRFP is 27 kD, while the predicted size of the three TagRFP fusion proteins is 68 kD. Potential degradation products for each RFP fusion are noted with asterisks.

We confirmed that the three TagRFP constructs are expressed as full-length fusion proteins in conidia using Western analysis with an RFP antiserum (Fig. 7). TagRFP is 27 kD and each Gα is approximately 41 kD. Correspondingly, each of the three Gα fusion proteins migrated close to their predicted molecular mass of 68 kD. Interestingly, although all three genes are expressed under control of the same promoter from a common genomic site, levels of GNA-1 are much higher than those of GNA-2 and GNA-3. This suggests some post-transcriptional regulation of Gα protein levels, with GNA-1 accumulating to higher levels in *N. crassa*. In addition, potential degradation products were observed for all three proteins (Fig. 7; asterisks), suggesting at least some of the variation in protein levels may be due to differences in protein turnover.

**Figure 8:**
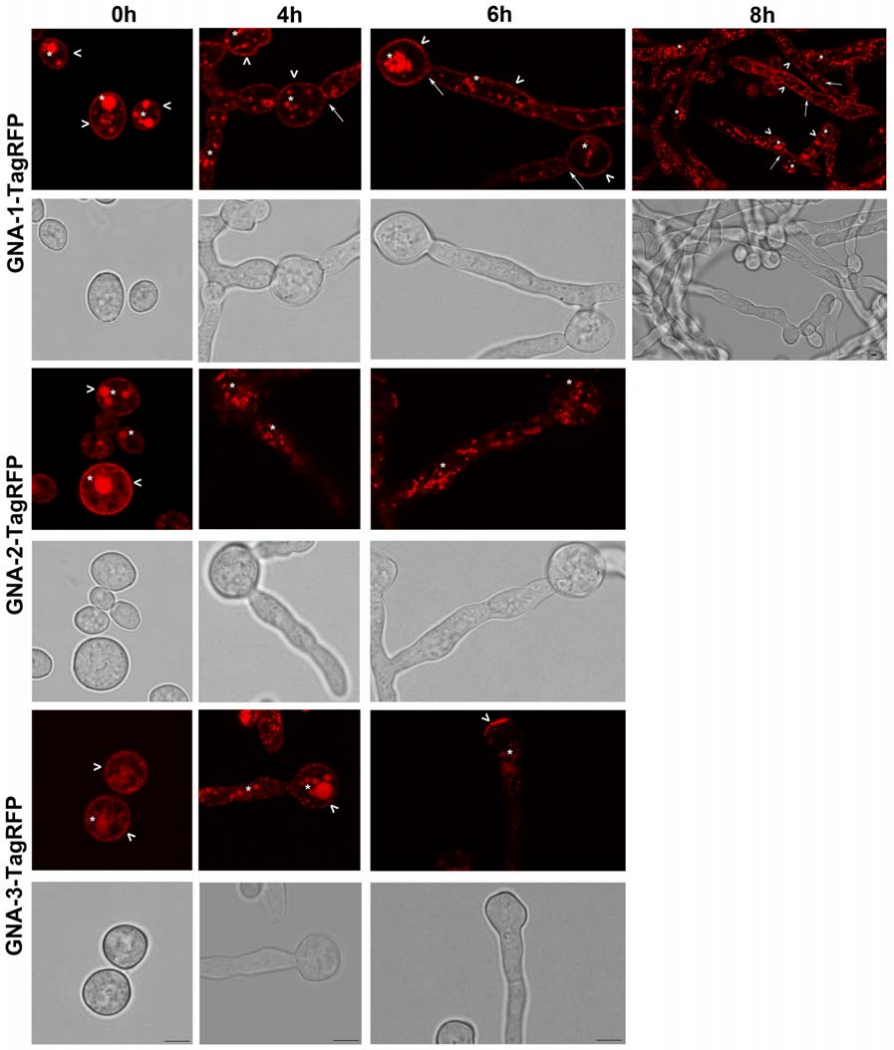
Localization of Gα proteins in germinating conidia. Conidia from strains expressing GNA-1-TagRFP, GNA-2-TagRFP, GNA-3-TagRFP and untransformed controls were inoculated on solid medium and analyzed after 0, 4, 6 and 8 h of growth. Images were captured by bright field and the 543 nm HyD laser using the Leica TCS SP5 II inverted confocal microscope. The arrowhead, asterisk and solid arrow correspond to plasma membrane, vacuole and septa localization, respectively. Panels are only shown for time points in which fluorescence can be detected above background. All panels are 4× zoom, with the exception of GNA-1 at 8 h, which is 2×. Scale bar = 5 µm.

**Figure 9:**
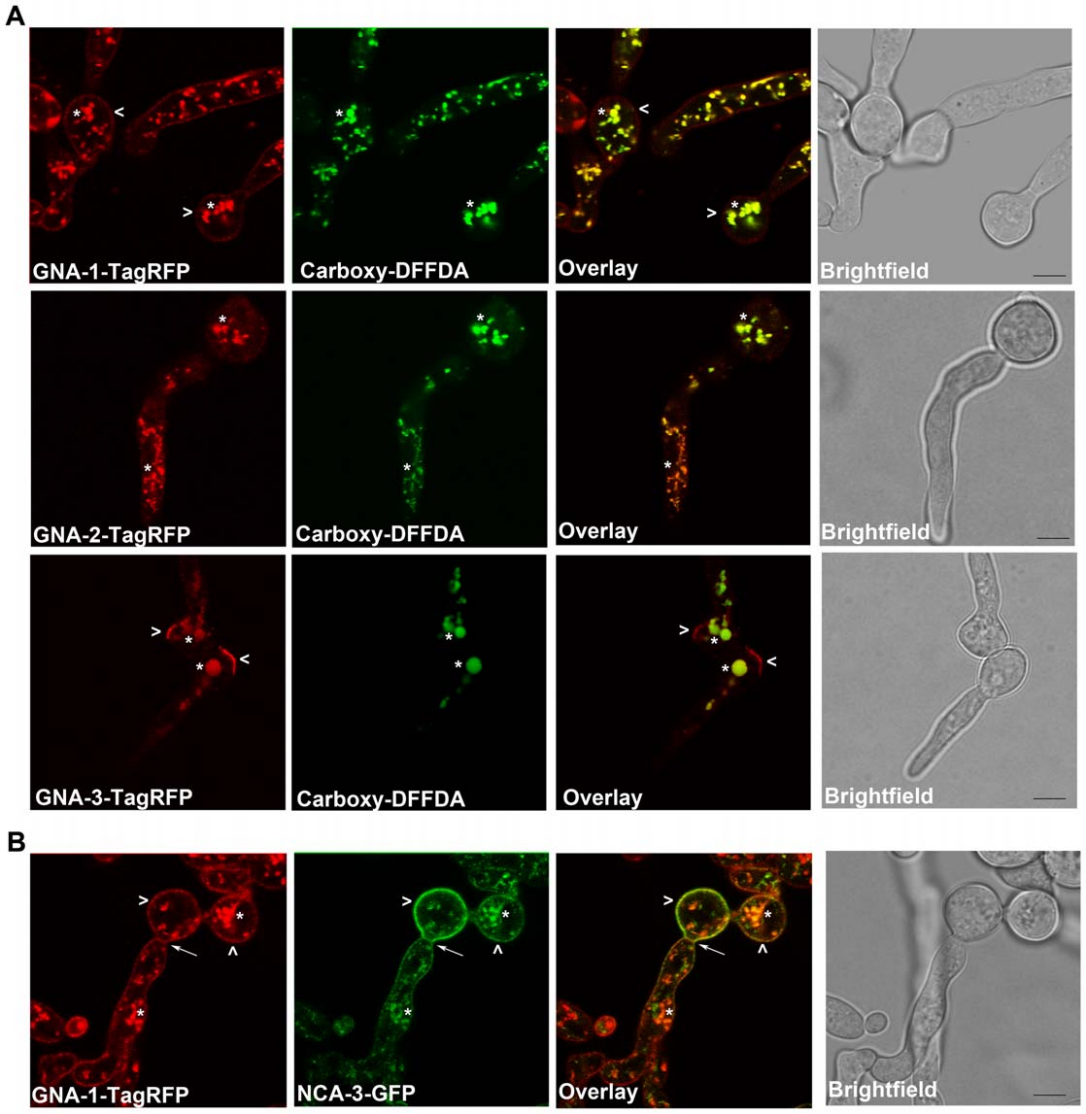
All three Gα proteins localize to vacuoles. **A.** Conidia expressing the corresponding Tag-RFP Gα were inoculated onto 100 mm solid VM at a concentration of 8×10^6^ conidia per plate. Plates were incubated at 30°C for 4 h (GNA-2 and GNA-3) or 6 h (GNA-1). The vacuolar dye Carboxy-DFFDA was applied at a concentration of 20 µg/ml. Images were captured using the Leica TCS SP5 II inverted confocal microscope. The RFP and GFP panels were merged to create the overlay. **B.** Conidia expressing both GNA-1-TagRFP and NCA-3-GFP were inoculated onto 100 mm solid VM at a concentration of 8×10^6^ conidia per plate. Plates were incubated at 30°C for 6 h. Images were captured using the Leica TCS SP5 II inverted confocal microscope. The RFP and GFP panels were merged to create the overlay. All panels are 4× zoom. Bar = 5 µm.

We examined the strains expressing TagRFP fusion proteins during conidial germination using confocal fluorescent microscopy (Fig. 8). Conidia were plated on solid medium and examined at four time points (0, 4, 6 and 8 h). At 0 h, all three Gα proteins localized to the plasma membrane and vacuoles (Fig. 8, 9A). The plasma-membrane localization is consistent with results previously observed during cell fractionation studies in our laboratory [2], [5], [6]; data not shown). The vacuolar localization of GNA-1-TagRFP, GNA-2-TagRFP, and GNA-3-TagRFP was validated by the overlapping signal observed between TagRFP and the carboxy-DFFDA vacuolar dye during confocal microscopy (Fig. 9A). In addition, a strain expressing a GFP-tagged protein, NCA-3, a Ca^2+^ ATPase known to localize to vacuoles and the plasma membrane, was combined with the GNA-1-RFP strain to produce a heterokaryon [19]. Many conidia and germinating hyphae from these heterokaryons exhibit green and red fluorescence, indicative of the presence of both NCA-3-GFP and GNA-1-TagRFP. Confocal microscopy of conidia from this heterokaryon confirmed that GNA-1 localizes to the plasma membrane and vacuoles (Fig. 9B). It is unclear whether the vacuolar fluorescence represents a functional localization or a site of protein turnover.

At 4 h of conidial germination, GNA-1 continued to display plasma membrane and vacuolar localization, and could also be observed on the first septum separating the conidium and the developing hypha (Fig. 8). In contrast, GNA-2 and GNA-3 were predominantly found in vacuoles, with less apparent plasma membrane localization (Fig. 8). At 6 h, GNA-1 was present in the plasma membrane, vacuoles and septa. (Fig. 8). GNA-2 exhibited a similar localization as observed at 4 h. Interestingly, in addition to vacuolar localization, GNA-3 was also found in distinct patches on the plasma membrane of the original conidium (Fig. 8). These patches were observed in 50% or more of the cells being sampled. At 6 h, the plasma membrane patches were even more easily observed in GNA-3-TagRFP strains, as the fluorescence in vacuoles and other regions of the plasma membrane began to weaken (Fig. 8). At 8 h, GNA-1-TagRFP fluorescence was still relatively strong in plasma membrane, vacuoles and septa (Fig. 8). In contrast, GNA-2- and GNA-3-TagRFP fluorescence was too dim to analyze in 8 h germlings (data not shown).

**Figure 10:**
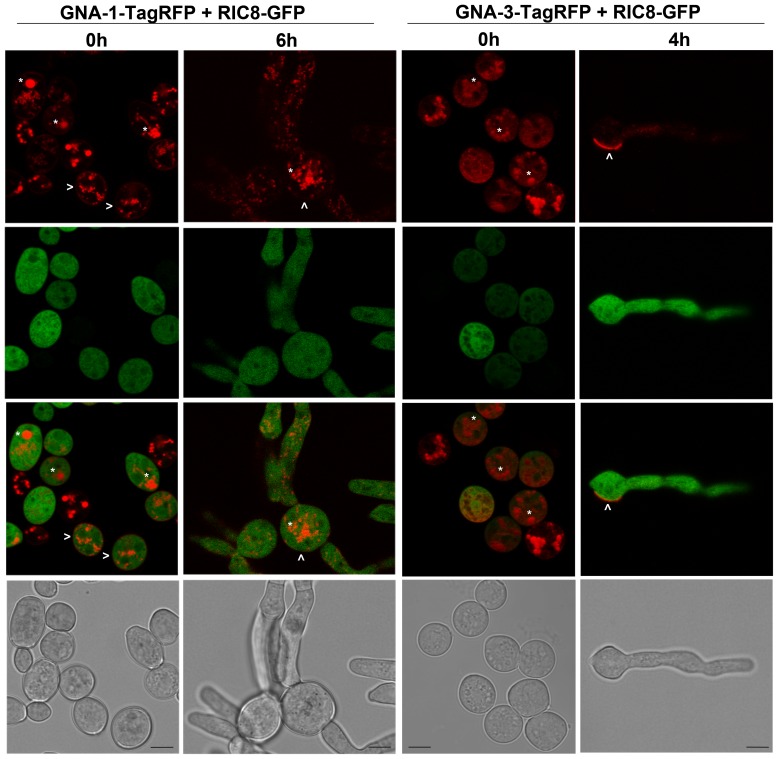
Localization of GNA-1-TagRFP and GNA-3-TagRFP with RIC8-GFP in germinating conidia. Conidia from a fused strain expressing GNA-1-TagRFP or GNA-3-TagRFP and RIC-8-GFP were harvested as described in the Materials and Methods. An aliquot containing 8×10^6^ conidia was spread on a 100 mm VM solid medium plate. Images were captured using a Leica TCS SP5 II inverted confocal microscope. Conidia were imaged immediately after inoculation on the solid medium plate. Conidia were allowed to germinate for 6 h or 4 h at 30°C before imaging. The first two panels were merged to create the third panel. All panels are 4× zoom. Scale bar = 5 µm.

We have previously demonstrated that RIC8 exhibits cytosolic localization in conidia and mature hyphae using fluorescent microscopy with an inverted compound microscope [9]. In this study, we utilized the more discriminating method of confocal fluorescence microscopy to explore possible co-localization of GNA-1-TagRFP and/or GNA-3-TagRFP with RIC8-GFP in cellular compartments during conidial germination. For these experiments, we cultured strains carrying the RIC8-GFP and GNA-1-TagRFP constructs together to produce heterokaryons expressing both fluorescent proteins. Similar to previous results, RIC8-GFP is cytosolic and excluded from vacant areas that appear to be vacuoles (Fig. 10). In contrast, GNA-1-TagRFP is located in the plasma membrane and vacuoles (Fig. 10) and the merged image did not reveal co-localization with RIC8-GFP in conidia or young germlings (Fig. 10). Similarly, co-localization was also not observed between GNA-3-TagRFP and RIC8-GFP (Fig. 10). In addition, there was no evidence for co-localization between the GNA-3 fluorescent patches and RIC8 (Fig. 10).

## Discussion

G proteins regulate nearly every facet of growth and development in fungi. During asexual development, in particular, they have been found to regulate both the timing and level of conidiation (rev. in [29], and in recent years have also been shown to regulate conidial germination [30], [31], [32], [33], [34]. Here, we report evidence showing that in *N. crassa* G protein signaling regulates both conidial development and germination, and this is regulated through the non-receptor GEF RIC8.

We observed that loss of any single G protein subunit has no effect on conidial morphology in *N. crassa*. However, loss of both *gna-1* and *gna-3* Gα genes leads to a dramatic increase in arthroconidia formation, which is also observed in the Δ*ric8* mutant. Arthroconidia are formed by the fragmentation of vegetative hyphae [23] and their production is proposed to be a default pathway in mutants defective in macroconidiation [24]. Expression of constitutively active (GTPase deficient) GNA-1 or GNA-3 in the Δ*ric8* mutant background does not lead to reduced arthroconidiation, suggesting RIC8 controls arthroconidia formation through Gα-dependent and independent mechanisms. While the exact mechanism by which RIC8 and the Gα proteins negatively regulate arthroconidiation is unclear, it may involve the MAK-1 MAP kinase signaling pathway, as deletion of any of the three kinases of this pathway leads to an overproduction of arthroconidia [35]. Additionally, loss of *rgb-1*, homologous to the B subunit of type 2A Ser/Thr phosphatases, also leads to production of large amounts of arthroconidia, perhaps through a negative effect on the MAK-1 pathway [24]. Interestingly, in spite of the requirement for G protein signaling genes during conidial development and germination, Δ*ric8* and all G protein subunit single and double mutants were found to produce conidial anastomosis tubes from macroconidia. However, it remains unclear whether the conidia from these mutants that do not germinate are also unable to produce conidial anastomosis tubes.

Conidial germination was found to be significantly reduced in the Δ*gna-3* mutant, indicating GNA-3 regulates conidial germination in *N. crassa*, as has been reported for homologs in *Penicillium marneffei* [34], *Aspergillus nidulans* [30] and *Botrytis cinerea* [31]. The germination defect is exacerbated in the Δ*gna-1* Δ*gna-3* double mutant, revealing a role for GNA-1 in the absence of GNA-3. GNA-1 homologs have been shown to regulate conidial germination in other fungi [32], [33], [36]. Germination is reduced in the Δ*gna-1* mutant relative to wild type, but this is only statistically significant 6 h after inoculation, consistent with GNA-1 playing a more minor role in *N. crassa*. The observation that GNA-3 is more important during germination in *N. crassa* than GNA-1 is interesting, given the Δ*gna-1* mutant displays a greater reduction in hyphal extension rate than the Δ*gna-3* mutant [2], [3]. This suggests that GNA-1 is more important for overall extension of hyphae rather than for early events during germination, while the converse is true for GNA-3. Germination of the Δ*gna-2* mutant is no different than wild type and germination is similar in Δ*gna-1* Δ*gna-2* Δ*gna-3* triple and Δ*gna-1* Δ*gna-3* double mutants, implying that GNA-2 is not required for conidial germination in *N. crassa*.

Our results demonstrate that the non-receptor GEF RIC8 regulates conidial germination primarily through the GNA-3 Gα subunit, with minor contribution from GNA-1. This observation is consistent with results from GTP binding assays demonstrating that RIC8 exhibits greater GEF activity towards GNA-3 than GNA-1 [9]. Interestingly, in *M. oryzae* loss of *MoRIC8* does not affect conidial germination [7]. Instead, MoRIC8 is required for normal asexual growth, conidiation, appressorium formation and pathogenicity [7].

It is likely that RIC8 and the Gα proteins regulate conidial germination in *N. crassa* through modulation of cAMP levels [3], [9], [37], [38]. The *N. crassa* Δ*ric8* mutant has decreased adenylyl cyclase protein and the Δ*ric8* mutation can be suppressed by a mutation in the protein kinase A regulatory subunit [9]. A mutation in the regulatory subunit leads to hyperactivation of the PKA catalytic subunit, thereby bypassing the need for wild-type levels of adenylyl cyclase. The Gα subunit GNA-3 is required to maintain wild-type levels of adenylyl cyclase, further supporting a role for G proteins in cAMP-dependent conidial germination [3]. In *B. cinerea* and *A. nidulans* cAMP plays a key role in conidial germination regulated by GNA-3 homologs [31], [39].

In this study we pioneered the use of an internal Gα tagging method in filamentous fungi based on a successful approach used in *Dictyostelium* and mammalian cells, inserting TagRFP into a conserved loop region in the Gα subunits [10], [11]. Using this approach we were able to localize GNA-1, GNA-2 and GNA-3 during conidial germination, revealing that all three Gα proteins localize to the plasma membrane in ungerminated conidia and young germlings and that GNA-1 can be detected in septa in older hyphae. All three Gα subunits were also detected in vacuoles, however, it is unclear if this is a functional localization, as proteins are often targeted for degradation by the vacuole [40]. Perhaps the most intriguing finding was the observation of patches of GNA-3-TagRFP on the plasma membrane at later time points during germination of conidia. This change in localization during the transition from conidium to germling may be related to the requirement for GNA-3 during conidial germination; further analysis is necessary to explore this hypothesis.

Using purified proteins in *in vitro* assays, we have previously demonstrated that RIC8 can bind to and act as a GEF for GNA-1 and GNA-3 [9]. However, confocal microscopy of strains expressing GNA-1-TagRFP or GNA-3-TagRFP and RIC8-GFP did not reveal any evidence for co-localization of either Gα protein with RIC8 in conidia or hyphae, suggesting the interaction may be transient. Studies in *Drosophila* neuroblasts have demonstrated cytoplasmic localization of Ric-8, while Gα_i_ was found in the apical cortex [41]. Ric-8 was also observed in ‘spot-like’ structures close to the apical cortex that partially colocalized with Gα_i_, indicating that their interaction may take place on the cytoplasmic face of the plasma membrane or in the cytoplasm [41].

Interestingly, the GNA-1-TagRFP signal was stronger than that of GNA-2 and GNA-3, and detectable over a longer period, suggesting this protein fusion may be more stable. This conclusion is also supported by the results from western analysis using RFP antibodies. In yeast, the Ste2p GPCR is ubiquitinated and targeted to the vacuole for degradation [42]. Analysis of *N. crassa* GNA-1, GNA-2 and GNA-3 using UbPred (http://www.ubpred.org/) predicts that GNA-1 and GNA-2 contain potential ubiquitination target sites, which may explain the vacuolar localization of these proteins. Future studies will explore whether this vacuolar localization is functional, and if so investigate the importance of G protein signaling in the vacuole.
