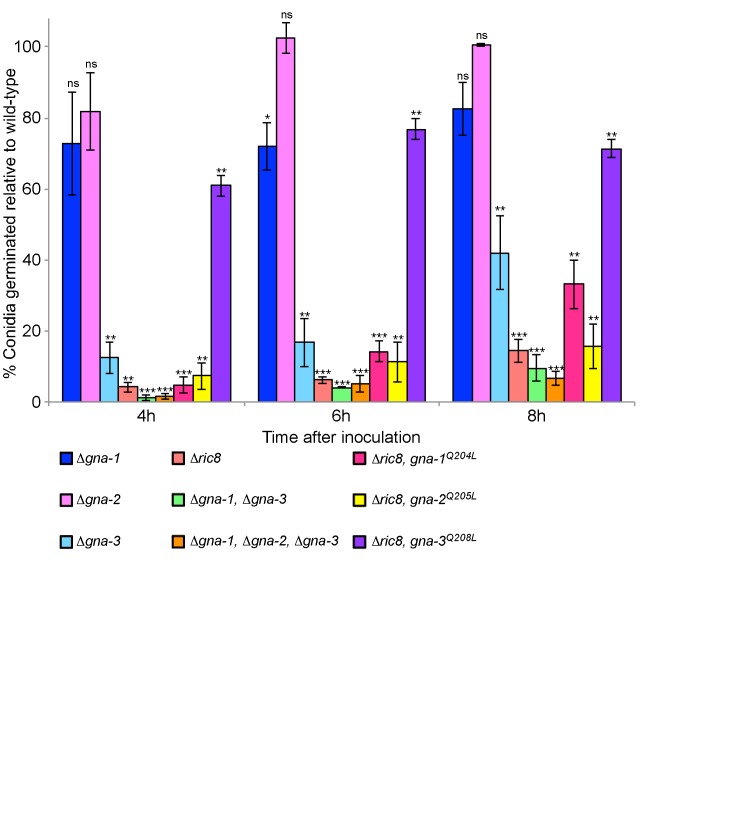# Correction: The Guanine Nucleotide Exchange Factor RIC8 Regulates Conidial Germination through Gα Proteins in *Neurospora crassa*

**DOI:** 10.1371/annotation/83a5d99e-3072-4bbf-a41b-08118aa77221

**Published:** 2013-03-14

**Authors:** Carla J. Eaton, Ilva E. Cabrera, Jacqueline A. Servin, Sara J. Wright, Murray P. Cox, Katherine A. Borkovich

The order of images (not the legends) for Figures 1-6 is incorrect. Figure 6 image should be Figure 1; Figure 1 image should be Figure 2; Figure 2 image should be Figure 3; Figure 3 image should be Figure 4; Figure 4 image should be Figure 5; Figure 5 image should be Figure 6.

The correct Figures are as follows:

Figure 1: 

**Figure:**
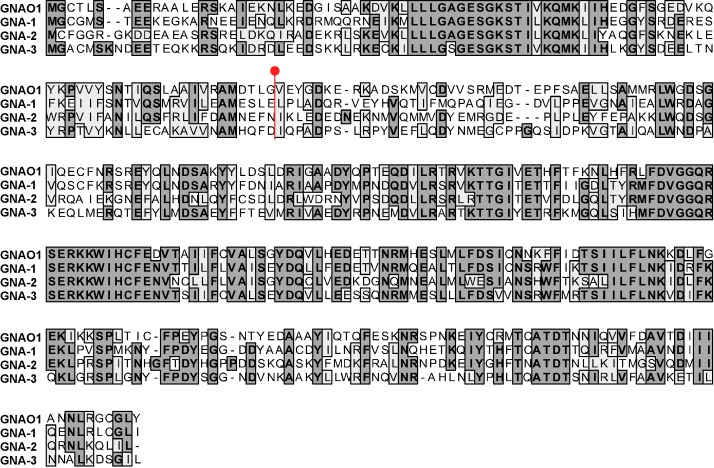


Figure 2: 

**Figure:**
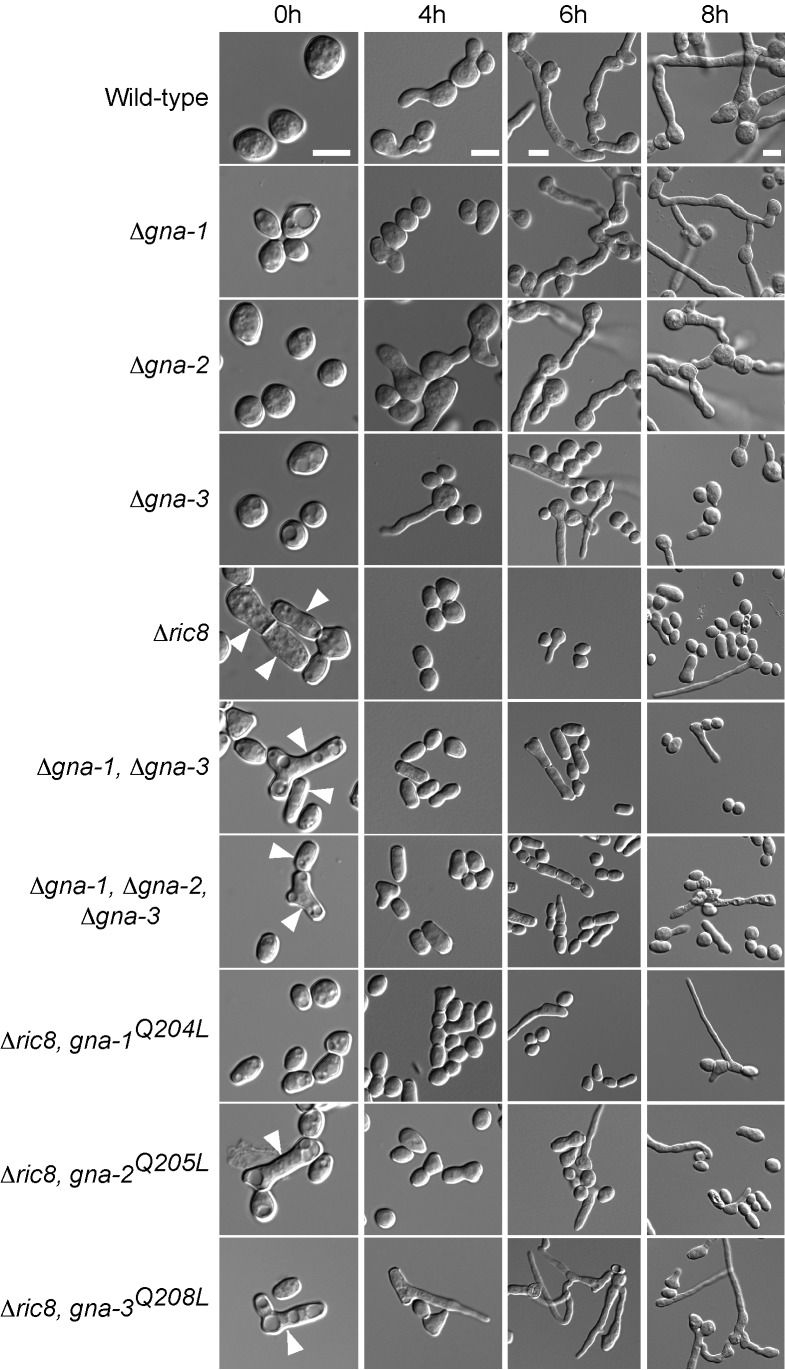


Figure 3: 

**Figure:**
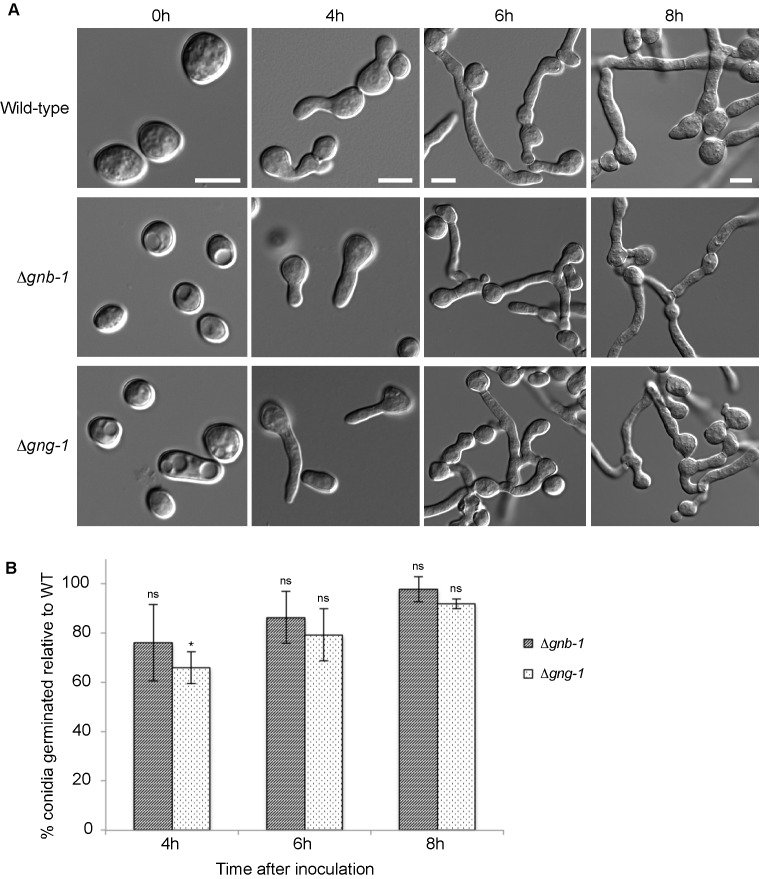


Figure 4: 

**Figure:**
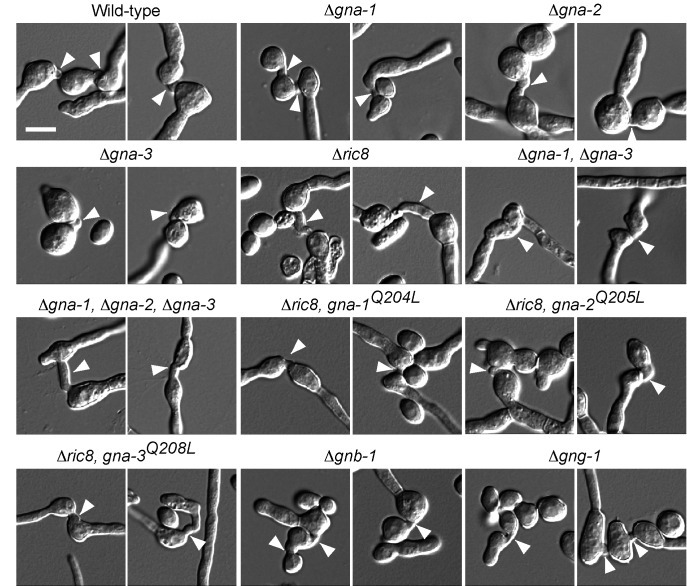


Figure 5: 

**Figure:**
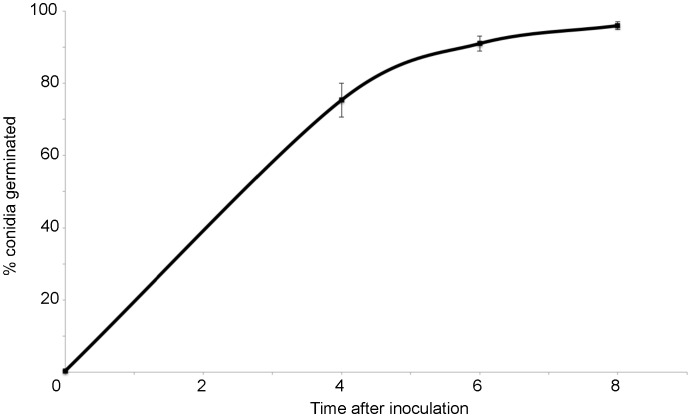


Figure 6: 

**Figure:**